# Policy actors’ perspectives on improving federal grants to promote the implementation success of evidence-based behavioral health practices

**DOI:** 10.1186/s43058-026-00882-6

**Published:** 2026-03-06

**Authors:** Blanche Wright, Grace M. Hindmarch, Jin Kim, Sarah B. Hunter, Danielle Schlang, George Timmins, Gregory A. Aarons, Jonathan Purtle, Alex R. Dopp

**Affiliations:** 1 Department of Psychology, University of Oregon, 1451 Onyx Street, Eugene, OR 97403 USA; 2 RAND, 1776 Main St, Santa Monica, CA 90401 USA; 3 Department of Health Policy & Management, University of California, Los Angeles, 650 Charles E. Young Dr. South, Los Angeles, CA 90095 USA; 4 School of Interactive Computing, Georgia Institute of Technology, 85 5th St NW, Atlanta, GA 30332 USA; 5 Department of Psychiatry and Altman Clinical and Translational Research Institute Dissemination and Implementation Science Center, University of California San Diego, 9500 Gilman Dr. (0812), La Jolla, San Diego, CA 92093 USA; 6 Department of Public Health Policy & Management and Global Center for Implementation Science, New York University School of Global Public Health, 708 Broadway, New York, NY 10003 USA

**Keywords:** Financing, Government, Evidence-based practice, Policy

## Abstract

**Background:**

To promote high-quality behavioral health service delivery, federal agencies often invest in evidence-informed and evidence-based practice (EBP) implementation through discretionary (i.e., competitive) grants. However, gaps remain in understanding how federal grant mechanisms can lead to large-scale reach (i.e., the extent of EBP integration into service systems). To understand EBP reach through federal grant mechanisms and provide actionable data, we conducted a series of focus groups with relevant federal and state policy actors to gather their perspectives on how to improve federal grants to support EBP implementation.

**Methods:**

This study was informed by ongoing research examining implementation outcomes (i.e., provider-level reach) from federal grants supporting the delivery of the Adolescent Community Reinforcement Approach (A-CRA), an EBP to address youth substance use. We conducted four focus groups, two with staff from state agencies who received federal A-CRA grants (*n* = 12) and two with U.S. federal agency officials responsible for behavioral health grant making (*n* = 12). We used the policy-adapted EPIS (Exploration, Preparation, Implementation, Sustainment) framework, conceptualizing grants as bridging factors between the outer policy context and inner service provision context. We used directed content analysis to characterize participant perspectives on contextual influences of EBP reach, and thematic analyses to identify how to improve federal grants for EBP implementation success (e.g., high levels of reach). We codified themes by EPIS domains and phases, and by policy actors.

**Results:**

We identified three contextual influences in Bridging Factors, six in the Inner Contexts, nine in the Outer Contexts, and three in Innovation characteristics. We found seven themes about grant improvements spanning all EPIS phases; examples include strengthening collaboration between multilevel actors in the implementation process (specifically federal and state agencies and treatment organizations), and integrating EBPs into insurance billing practices (which also included insurance leadership as a key actor group).

**Conclusions:**

To support large-scale EBP reach, federal funders may consider using implementation strategies that foster greater collaboration between policy actors from multiple sectors and organization levels. This work also demonstrates how implementation science frameworks can be used to study the influence of federal financing initiatives for EBPs in the area of youth behavioral health.

**Supplementary Information:**

The online version contains supplementary material available at 10.1186/s43058-026-00882-6.

Contributions to the literature

- Findings begin to fill a gap in implementation science around understanding how to improve federal grants in support of key implementation outcomes like reach.
- This study contributes to a limited literature about actions that policy actors across multiple system levels can take during federal grant design and implementation to maximize EBP reach, and is informed by the perspectives of key actors.
- Study results offer real-world implications for various actors in federal and state government, as well as service delivery organizations, and highlights bridging factors and interorganizational alignment, which are understudied in implementation science.

## Introduction

Evidence-based practice (EBP) implementation can be costly and complicated, involving administrators, supervisors, providers, and other staff [1]. Prior literature has identified grants from governmental and private organizations as key strategies for financing EBP implementation, and several implementation strategies related to grant funding have been established [2–5]. In the United States, federal agencies have historically used discretionary (i.e., competitive) grants to invest in EBP implementation, aiming to promote delivery of high-quality, publicly funded behavioral health services. In discretionary grants, federal agencies facilitate a competitive proposal process and use their discretion to select recipients [6]. For example, in fiscal year 2024, Congress allocated $7.45 million dollars in discretionary funds to the Substance Abuse and Mental Health Services Administration (SAMHSA) [7]. SAMHSA administrators decided how to disburse these funds to support public behavioral health systems and service recipients and often used discretionary grants to allot funds for specific target populations and/or for specific EBPs. When applying the Exploration, Preparation, Implementation and Sustainment framework (EPIS) [8, 9], such federal grant mechanisms can be conceptualized as bridging factors that link the federal policy context (i.e., “outer” context) to the “inner” context where the treatment is delivered through service provider or treatment organizations [10, 11].

Despite their role in funding, little is known about how the design of federal grant mechanisms, involved parties, and contextual factors influence behavioral EBP implementation success [2, 12, 13]. Researchers have described that federal government agencies encourage EBP delivery through legislative policy and funding requirements [5, 14], and some recent studies have focused on how state and local funding policies can affect the reach of behavioral health EBPs [15–19]. However, research on federal EBP grant funding and implementation outcomes is sparse despite consistent acknowledgement that changes in governmental infrastructure are needed to scale EBPs [20, 21]. One qualitative study with stakeholders involved in a federal grant to implement Trauma-Focused Cognitive Behavioral Therapy identified funding as an influential factor on implementation outcomes, including support for changing the insurance billing infrastructure so EBP activities can be reimbursed [22]. Particularly for substance use treatment, studies have examined how EBP terminology is embedded in federal grant announcements [13, 21]. A prior study examined whether substance use disorder treatment organizations were more likely to obtain federal funding if they used EBPs and found mixed results, exposing a gap between practice and government goals [23].

To efficiently scale EBPs, it is important to understand how federal grants are associated with markers of EBP implementation success, such as reach (i.e., integration of an EBP into a service system) [24]. A recent study compared EBP provider-level reach (i.e., proportion of EBP-trained clinicians) between two SAMHSA grant types and found that grants awarded to state substance use service agencies resulted in lower provider-level reach rates than those awarded directly to substance use treatment organizations – the opposite of what was intended in the state-level grant design [25]. Qualitative results provided context to these unexpected results, as greater perceived strategic planning and SAMHSA oversight were more salient facilitators in grants awarded directly to treatment organizations. Still, it is unclear how to improve grant-making activities across EPIS phases to ensure EBP implementation success.

Furthermore, greater specificity in understanding individuals’ roles in designing and carrying out policy-level implementation strategies (e.g., federal grants) can help close research-policy-practice gaps [26]. Often, implementation researchers have used “policymaker” as a broad term encompassing elected officials (e.g., state legislators; governors), appointees (e.g., director of executive branch government agencies, like SAMHSA), and their staff at a variety of levels [27, 28]. Such a broad term may have been used because policy has been understudied in implementation science [29–31], thereby leaving a conceptualization gap. To refine conceptualizations of policymakers within the field, implementation researchers have been encouraged to apply an established taxonomy distinguishing between “Big P” and “little p” policies [9]. “Big P” policy generally refers to outer context formal laws, rules, or regulations whereas “little p” policy refers to inner context organizational rules [32, 33]. Both policymakers (i.e., elected or appointed officials) and agency employees are *policy actors*—that is, anyone who is involved in designing, adopting, preparing, implementing or sustaining policy [26]. Applying this taxonomy to federal grant mechanisms, *policymakers* often help create “Big P” government policy (e.g., approving a grant funding announcement or allocating funds to a priority area for grants), and *agency employees* often enact the policy and carry out policy-related administrative tasks using “little p” procedures or guidelines for activities such as soliciting grant applications, awarding funds, and managing funded awards; however, both types of policy actors can be involved in both levels of policy. The EPIS framework can help articulate the complex interplay of key roles, multilevel contexts, and contextual factors [9], including delineating *when* in the EBP implementation process policy actors need to take actions.

Overall, there is a need to identify actionable ways to improve federal grants for EBP implementation success and sustainment. To help address this gap, our qualitative study explored federal and state agency officials’ perspectives on how to improve federal grants for successful EBP implementation. We applied the policy-adapted EPIS framework [9] to identify and characterize contextual influences to achieving EBP reach based on two types of federal grants (i.e., state level and organization level), and identified themes characterizing actions that policy actors can take to improve federal EBP implementation grants, specifying relevant policy actors and EPIS phase(s).

## Methods

### Study context

This study was part of a larger research project comparing implementation outcomes of an EBP for adolescent substance use, the Adolescent Community Reinforcement Approach (A-CRA), between two SAMHSA grant mechanisms. A detailed study protocol is available [34], so only details relevant to the current study are discussed here. The study was approved by the RAND IRB (Protocol #2020-N0887). We describe this study following the Consolidated Criteria for Reporting Qualitative Research (COREQ) checklist (see Additional File 1).

The two SAMHSA grant types were: (1) “organization-focused” grants awarded directly to treatment organizations that implemented A-CRA, and (2) “state-focused” grants awarded to state substance use service agencies, which were tasked with creating statewide A-CRA training and implementation infrastructure to support treatment organizations. We initiated the focus groups after the aforementioned finding that organization-focused grants led to higher provider-level reach than state-focused grants [25].

### Procedures and participants

We conducted two sets of focus groups with two policy actor groups, one set with federal agency staff and one set with state agency staff. We used purposive sampling to recruit participants via email.

For federal officials, we identified potential participants through our professional networks; we used snowball sampling by asking officials to nominate others who could be invited to participate, and participation of multiple officials per agency was permitted. We invited 22 federal officials; 13 agreed to participate, three declined, and six were not included for logistical reasons (e.g., unavailable on selected dates). We prioritized agencies within the U.S. Department of Health and Human Services as they are the primary federal funder of EBP implementation grants; all but one of the participating federal officials represented four such agencies, and the last participant also worked in the area of state health policy. Their roles broadly related to federal grants for EBPs in health and social services, including director-level roles that oversaw grants and analyst-level roles that supported execution of awarded grants.

For state agency officials, we invited individuals who had previously participated in the larger project due to their agency having received a state-focused A-CRA grant from SAMHSA [34]. We sought to recruit a geographically diverse sample, and thus required that each participant worked in a different state. We invited 41 state officials; 14 agreed to participate, six declined, two did not respond, and 19 were not included for logistical reasons. Although tribal government and territories also receive federal grants, they were not included in our sample because none were recipients of the A-CRA SAMHSA grants being studied. State officials came from a variety of state-level agencies and divisions/departments working on adolescent and/or substance use services (e.g., child and family services, behavioral health services). Most worked as staff managing A-CRA state-focused grants, but some worked in Division or Department leadership or other roles (e.g., evaluator).

Overall, of the individuals invited, 43% (27/63) agreed to participate, and 89% of those who agreed (24/27) participated in a focus group. We conducted four virtual focus groups with 5–7 participants each via the secure Zoom for Government platform in Fall 2023. Each focus group lasted 60 min; two groups were with state officials (*n* = 12 from 12 states) and two groups were with federal officials (*n* = 12 from five agencies). AD facilitated the focus groups with support by BW, DS, or SH, and obtained verbal informed consent from participants. We audio-recorded focus groups and produced de-identified transcripts for analysis. Participants were compensated with a $100 gift card; however, some participants declined compensation as they saw participation as part of their job or had restrictions about accepting external payment. To protect the privacy of the officials, we did not collect demographic information.

#### Focus groups

To facilitate discussion, we asked participants to review a one-page research summary prior to the focus group that emphasized the role of EBP funding and summarized our findings comparing organization-focused and state-focused SAMHSA grants as a case example [25, 34]. Additional File 2 presents the summary. Next, the facilitator asked questions from a focus group guide that was based on the policy-adapted EPIS framework [9]. Participants were asked to discuss their perspectives on improving federal grants for EBP implementation, drawing on the comparison from the case example while also encouraging broader discussion of other EBPs and federal grant types. The facilitator used prompts and follow-up probes to encourage discussion of how factors across EPIS domains could influence the success of federal EBP implementation grants: (1) political and community factors, including federal agency funder characteristics (Outer Context); (2) state agency and service delivery organization implementer characteristics (Inner Context); (3) grant mechanism design and implementation, including EBP implementation support and monitoring processes (Bridging Factors); and (4) EBP characteristics (Innovation). See Additional File 3 for the focus group guide.

#### Researcher reflexivity

Per COREQ, we describe coding team characteristics that informed our perspective on the findings. AD, BW and SH (researchers) hold PhDs and DS (survey manager) has a MA; all drew on their experience with implementation research and practice for mental health and substance use services to guide focus group discussions. Similarly, GMH and JK (research assistants) held Bachelor’s degrees and received training in those topics prior to assisting BW with analysis. All team members have experience examining federal financing strategies influence on the reach of A-CRA [25]. BW, SH, DS, GMH, and JK identify as female, and AD identifies as male.

### Analysis

BW, GMH and JK conducted qualitative analyses using Excel and NVivo, with input from the other co-authors. We took a stepwise approach to identify qualitative codes and themes from the transcripts. Initially, we used directed content analysis to identify contextual influences (i.e., barriers and facilitators) on using federal grants to implement EBPs for behavioral health. Organization of the contextual influence codes was guided a priori by the EPIS domains: outer context, bridging factors, two inner contexts (i.e., (1) state agency and (2) service delivery organization), and innovation. We discussed discrepancies between coders to achieve consensus. Although we sought to first identify implementation barriers and facilitators, most of the contextual influences were considered both a barrier and facilitators to using federal grants for EBP implementation, so we decided to further interpret the data to identify more actionable findings.

Specifically, we conducted a thematic analysis following guidance from Braun and Clark [35] across all focus groups to identify cross-cutting recommendations for improving federal EBP implementation grants. As we completed the thematic analysis, we iteratively reviewed the barrier/facilitator contextual influence findings to ensure we did not overlook key information. Thematic analysis allowed for both an inductive approach, in which we developed themes bottom-up from the data, and a deductive approach that drew on the contextual influence codes from step one and categorized themes using EPIS [35]. For each theme, we reviewed the relevant transcript excerpts and coded each excerpt with applicable EPIS domain(s) and phase(s); the phases were operationalized in relation to the grant-funded EBP implementation process. Exploration (i.e., selection of an EBP for implementation) refers to activities prior to a federal grant being awarded. Preparation refers to activities taking place prior to EBP implementation but after the grant was awarded. Implementation refers to EBP training activities and active delivery. Sustainment refers to activities taking place after the federal grant funding ended. Finally, the inductive aspect of analysis also included characterization of policy actors [9] as we could not find an existing coding system that effectively described the various actors we identified. In sum, each theme describes important actions, when those actions take place, and the *policy actors* whose involvement and/or decision-making power would be needed to enact the actions.

To support the overall coding and theme identification processes for the main analyses, we developed and iteratively updated a codebook that outlined codes, EPIS phases and domains, and policy actors. The coding team checked themes from all sources across both federal and state officials until themes were “coherent, consistent and distinctive” [35]. We updated the codebook after discussions and when discrepancies between coders were clarified. A version of the thematic analysis was published as policy recommendations (with real-world examples) in a report [36], but no other components of the analysis were included in that publication.

## Results

Contextual influences (i.e., barriers and facilitators) on EBP implementation success within the context of federal grants were identified in all EPIS domains. Most of these contextual influences were described as both a barrier and facilitator depending on circumstances. Although the duality of factors limits the actionability of findings, three higher-level patterns were clear. First, within the Inner Context, state agency and treatment organization personnel were key influences when pursuing EBP reach especially within the context of federal funding; participants discussed the ideas of EBP “champions” and offering provider incentives. By contrast to Inner Context personnel, in the Outer Context, the influence of individuals who worked in federal agencies was mentioned less often, which may suggest reduced influence. Second, we conceptualized federal grant mechanisms as formal partnership arrangements between federal, state, and treatment organizations actors – that is, Bridging Factors linking Outer and Inner contexts. Results indicated that these arrangements were major influences (i.e., how requirements in grants impacted EBP implementation) and could facilitate EBP reach if they were general enough to be tailored to the local context. When federal grant arrangements required interorganizational partnership (e.g., state agencies with community-based treatment organizations), the success of the coordination and collaboration were contingent on relationships and shared commitment. Third, the culture and climate of treatment organizations, state agencies, and federal agencies were each considered influential, with a duality in which it was a facilitator to have organization/agency buy-in and commitment to pursuing/offering federal funding for EBPs; however, the processes could be barriers as they were perceived to be “flawed,” cumbersome, and “require sophistication” to apply to federal EBP grants. Additional File 4 summarizes the contextual influences in greater detail, including factors in the Innovation domain (i.e., A-CRA characteristics), and provides exemplar quotes for the most common barriers and facilitators only.

To support actionability, the main results were seven cross-cutting themes about challenges with federal grants and ways to improve grants for EBP implementation success. All themes included perspectives from federal and state officials. Participants primarily discussed EBPs generally, with some discussion of EBPs addressing substance use specifically. The following sections are organized by the EPIS phase that we considered most relevant to the actions described. Themes covered all four EPIS phases, and some themes cut across multiple phases. Table 1 summarizes the themes and provides characterizations by EPIS phase, EPIS domain factors, policy actors and example quotes. Table 2 provides definitions of the policy actors we identified in the data.

**Table 1:** Summary of cross-cutting themes codified by EPIS phase, EPIS domains and policy actors

**Phase**				**Cross-Cutting Theme**	**EPIS Domain: Factors **N/A = not applicable	**Policy Actors**	**Example Quote**^**a**^
**E**	**P**	**I**	**S**				
X				**Rigid or Variable Standards of Evidence Impact Consideration and Selection of EBPs**	**Outer Context**: Federal Agency Culture **Bridging Factor**: N/A **Inner Context 1 (State)**: State Agency Culture **Inner Context 2 (Treatment Org)**: N/A **Innovation:** Evidence base	- Federal Agency Leadership - State Agency Leadership	“In the child welfare world, the [prevention programs], evaluation, clearinghouse and rules around funding evidence-based programs, the experience there is more limited, and it has been spotty. We find that states are gravitating to a few programs that have much longer track records and higher levels of evidence and are less interested in some of the programs that are less well known, have less support technically. That they're going off for the name brand, a few of the name brand programs and have been less interested in developing additional models or evidence for other models that are of interest in the field but haven't met some of the evidence bars yet.”
X	X	X		**Importance of Building Capacity for Low-resourced Treatments Organizations to Obtain Federal Funding and Complete Grant Activities**	**Outer Context:** Federal Agency Policies **Bridging Factor:** Formal Grant Arrangements Fit with Context **Inner Context 1 (State):** State Agency Culture and Climate **Inner Context 2 (Treatment Org):** Treatment Organization Workforce Capacity Factors **Innovation:** N/A	- Federal Agency Leadership - State Agency Leadership - Treatment Organization Leadership - Federal Grant Reviewer	“I think that diversity and equity is really important, especially as we're looking at things like harm reduction and we're looking at money related to youth, especially moving on primary prevention and universal prevention. We're talking about very small, kind of grassroots organizations. So, getting money out to them, again, the more barriers to entry, so to speak, the harder it's going to be. And that really, I guess, cuts across for all grants.”
	X	X		**Bi-Directional Bridging Factors of Communication and Cohesion among Multilevel Collaborators Throughout Grant-Related Activities**	**Outer Context:** Federal Agency Policies **Bridging Factor:** Formal Grant Arrangements Fit with Context **Inner Context 1 (State):** State Agency Culture and Climate **Inner Context 2 (Treatment Org):** Treatment Organization Culture/Climate **Innovation:** N/A	- Federal Agency Leadership - State Agency Leadership - Treatment Organization Leadership	“We have some examples of where we really try to…knit together the state-level work with the community work so that they're not happening at cross purposes. I think that is often, if we have the resources and all the other things, I think that's where you get the sort of most bang for your buck, because then there's like a feedback loop moving up and down between states, the state and the community, and also can help with some of the other challenges like is this thing Medicaid reimbursable, is there like a state-level orientation of what's happening and what the barriers are, etc.?”
	X	X		**Grant Standards Need to Balance Fidelity and Flexibility for Tailoring EBPs**	**Outer Context: **Federal Agency Policies; Client Characteristics **Bridging Factor:** Formal Grant Arrangements Fit with Context **Inner Context 1 (State):** State Agency Culture and Climate **Inner Context 2 (Treatment Org):** Treatment Organization Workforce Capacity Factors **Innovation: **EBP Fit with Context; Evidence Base	- Federal Agency Leadership - State Agency Leadership - Treatment Organization Providers - EBP Developers	“There also often are elements of some of the evidence-based practices that communities either find it conflicts with their experience base or that they end up not having the full resources to do a program and so try to do it not with fidelity, but to pick and choose pieces of a practice that may or may not produce the same kinds of results. And I think we have a tension with wanting the evidence-based practice with fidelity monitoring and that comes with some of that stuff but then also the reality in communities…”
	X	X	X	**Workforce Capacity Issues Limit EBP Implementation Success**	**Outer Context: **N/A **Bridging Factor:** N/A **Inner Context 1 (State):** State Agency Workforce Capacity Factors **Inner Context 2 (Treatment Org):** Treatment Organization Workforce Capacity Factors **Innovation:** EBP Fit with Context	- State Agency Leadership - Treatment Organization Leadership - Treatment Organization Providers - EBP Developers	“It doesn't matter how great the model is. A lot of times there's just that we don't have the capacity to send people there or, if there's turnover, to retrain people and it's a whole lot easier if you use something that is used more throughout the entire state. …They're already trained in Motivational Interviewing so they can hit the ground running as opposed to something like A-CRA that is a little bit more specialized and it's a great, great program. That's why we put it in and implemented it for our grant, but it was still a lot of work for them to manage as well.”
	X	X	X	**Longer Grant Award Periods Can Support Implementation Success**	**Outer Context:** Federal Agency Policies **Bridging Factor:** Formal Grant Arrangements Fit with Context **Inner Context 1 (State): **State Agency Workforce Capacity Factors **Inner Context 2 (Treatment Org):** Treatment Organization Workforce Capacity Factors **Innovation:** EBP Fit with Context	- Federal Agency Leadership - State Agency Leadership - Treatment Organization Leadership - Treatment Organization Providers	“[Grants are] three to four years…there might not be adequate time to actually get started and implement and do evaluations. And certainly with research, it takes more time normally. And so I think the timeline for a program to implement EBPs or models is also an important factor and if there is not adequate time, it can hinder what we're trying to do in EBPs. And then also ensuring that there's resources available for sustainability after the program leaves.”
	X	X	X	**Support for Insurance Reimbursement of EBPs**	**Outer Context:** Influence of Government Policies-Medicaid **Bridging Factor:** Formal Grant Arrangements Fit with Context **Inner Context 1 (State): **N/A **Inner Context 2 (Treatment Org):** Treatment Organization Policies **Innovation:** EBP Fit with Context	- Federal Agency Leadership - State Agency Leadership - Treatment Organization Leadership - Treatment Organization Providers - Insurance Leadership - EBP Developers - Federal and State Legislatures	“I think helping grantees to be able to do Medicaid billing is a big one and many of them are intimidated by that and don't want to tackle it. [EBPs] that are being discussed are Medicaid billable, that really is the way to sustain them.”

^a^To preserve participant confidentiality, we do not specify if quotes were from state or federal officials

**Table 2:** Policy actors identified in themes

Policy Actor	Description
Federal and State Legislatures	Federal and state policymakers that appropriate funds (e.g., to Medicaid)
Federal Agency Leadership	Federal agency administrators who have decision-making power
Federal Grant Reviewer	External reviewers of federal grant applications
State Agency Leadership	State agency administrators who have decision-making power
Treatment Organization Leadership	Treatment organization administrators who have decision-making power
Treatment Organization Providers	Treatment service providers working in treatment organizations (i.e., clinicians, clinical supervisors)
EBP Developers	Developers of treatment components but also certification processes and ongoing technical assistance
Insurance Leadership	Insurance, including Medicaid, administrators who have decision-making power

### Exploration phase themes

#### Rigid or variable standards of evidence impact selection of EBPs

Officials highlighted that federal agencies and states use various metrics of “evidence-based” which creates inconsistency when selecting EBPs for implementation grants. Selection of EBPs for federal grants occurs in the Exploration phase. Officials noted that certain treatments are favored for federal implementation grants and by state agencies due to an overreliance on the number of randomized controlled trials to establish an “evidence-based” status. They noted that relying on these standards creates a preference towards name-brand EBPs which excludes treatments and programs with other forms of evidence (e.g., mixed methods; qualitative; community values) from being chosen when designing implementation grants. The metrics used to evaluate the evidence base (innovation) also vary by federal agency (outer context) and state agency culture (inner context).

#### Importance of building capacity for low-resourced organizations to obtain and complete grants

For EBPs to be equitably disseminated, both state and federal officials identified the need to aid low-resourced organizations in building capacity to obtain grant funding, and when obtained, complete required activities. This was discussed by federal officials, who described organizations that were smaller, community-based, and/or served high-need populations (e.g., rural, asylum seekers, LGBTQ +) as having limited resources. Aiding low-resourced organizations to apply for grants would begin in the Exploration phase when federal grants are designed and applications are solicited. In addition, low-resourced organizations would benefit from federal personnel support when completing grant activities, which would occur in the Preparation phase (e.g., establishing fund transfer), and the Implementation phase (e.g., submitting progress reports). Providing support for organizations in grant applications would potentially require changing the federal agency policies (outer context), strengthening fit of formal grant arrangements with implementation contexts (bridging factor), and addressing gaps in treatment organizations’ workforce capacity (inner context). If federal grants are awarded to state agencies, shifts in state agency culture (inner context) may be needed to provide support to organizations. Bolstering grant capacity would require leaders from federal agencies, potentially state agencies, and the treatment organizations to collaboratively identify training or workforce needs and incorporate needs into the grant making process. Beyond federal agency leadership, federal grant reviewers may need training to help facilitate more equitable grantee assessment.

### Preparation and implementation phase themes

#### Bi-directional communication and cohesion among multilevel collaborators throughout grant-related activities

Officials emphasized importance of strengthening collaboration among federal agencies, state agencies, and treatment organizations during grant-funded activities i.e., bridging factor), especially when preparing for EBP implementation. Officials perceived that ongoing communication and building partnerships or relationships across federal, state, and service delivery-level organizational systems would help accomplish grant goals and promote EBP reach. There was some overlap with the previous theme about aiding low-resourced organizations in obtaining federal grants, given that it would entail some level of collaboration between federal and state agencies with treatment organizations. Strengthening collaboration would require actions once grants are awarded in the Preparation and Implementation phases as collaborative relationships must first be established and then further developed during active EBP delivery. Federal agency policies (outer context), state agency and treatment organization culture and climate (inner context) would likely need to shift if these entities are not accustomed to multilevel communication and collaboration. More collaboration during grant activities may necessitate changes to the formal grant arrangement (bridging factor) to structure communication or even require it.

#### Grant standards need to balance fidelity and flexibility for tailoring EBPs

Participants identified tension between EBP fidelity standards and the flexibility that providers need in order to tailor to their local service contexts (e.g., workforce capacity; client needs). Officials noted that fidelity standards are requisites to EBPs and embedded in some certification processes; they also noted that high fidelity standards are embedded in the evaluation that is often required in federal grants. Managing grant standards for fidelity with flexible EBP delivery would occur in the Preparation and Implementation phases; during Preparation, adaptations may need to be anticipated so they can be incorporated into Implementation. To balance fidelity and adaptation, federal agency policies may need to consider client characteristics (outer context) and the formal grant arrangement fit with service context (bridging factor) to determine whether adaptations should be expected, and how to evaluate EBP delivery quality with imperfect adherence. For state-focused grants, EBP fidelity expectations within state agency culture (inner context) may need to transform. Factors that may drive the need for flexible EBP delivery are treatment organization workforce capacity (inner context) and relatedly, the extent to which fidelity influenced the “evidence-base” for the EBP and ultimately, EBP fit with context (innovation). Making changes to how fidelity and tailoring are monitored and balanced in grant opportunities would require actions by federal and state agency leaders. Other key policy actors would be the treatment organization providers as they are interfacing directly with clients. The EBP developer can also play a crucial role, such as providing guidelines about which treatment adaptations are permissible to achieve desired clinical outcomes.

### Sustainment phase themes

#### Workforce capacity limit EBP implementation success

Many officials highlighted the various workforce capacity challenges that limit EBP reach, including negative attitudes towards EBPs. A notable challenge was staff turnover at state agencies and treatment organizations. Officials noted the overall provider shortage and high turnover of supervisors was partially driven by higher paying jobs elsewhere. Addressing workforce turnover would require action within the Preparation, Implementation and Sustainment phases with the overarching goal to facilitate EBP sustainment. Efforts to preserve the workforce may be planned during Preparation and enacted during Implementation, when provider workload often changes significantly. Relevant EPIS factors were state agency and treatment organization workforce capacity (inner contexts) and EBP fit with context (innovation) such that the workforce may not be allotted time to accomplish all training and certification requirements. State agency and treatment organization leaders may work independently or together to identify workforce capacity limitations and solutions in support of EBP reach and sustainment. They likely would benefit from integrating treatment organization providers perspectives into decisions. Steps to address turnover could potentially involve the EBP developer if the training and certification requirements are extensive and make it challenging to train new providers.

#### Longer award periods can support implementation success

Many officials discussed that lengthening grant award periods can strengthen potential for successful EBP implementation and sustainment. Participants described that extended time would allow for more planning for the active EBP implementation, as well as time to complete and submit required evaluations. In one focus group, a participant described the benefits of continued technical assistance after grants ended to support EBP sustainment. Longer grant awards would translate as longer Preparation and Implementation phases, and ultimately, are relevant to the Sustainment phase given that the motivation for more time is to maximize EBP impact. Lengthening of grant award periods requires changes in federal agency policies (outer context) and the formal grant arrangements (bridging factor) to adequately account for the extra time that the state agency and treatment organizations’ workforces (inner context) need to accommodate EBP delivery (innovation). Federal agency leadership has the decision-making power to lengthen grant periods. Leaders of state agencies and treatment organizations will need to prepare for the EBP implementation at a pace that makes it more feasible to be successful during the implementation and sustainment phases.

#### Support for reimbursement of EBPs

The final theme was the integration of EBPs into insurance billing, especially Medicaid, so that organizations could get reimbursed for EBP activities in pursuit of better reach and sustainment. Initial integration would require planning within the Preparation phase but would also necessitate maintenance within the Implementation and Sustainment phases. Relevant factors are government policies related to Medicaid (outer context), formal grant arrangements (bridging factor), treatment organization policies (inner context), and the EBP’s fit with the funding context (innovation). Leaders of federal and state Medicaid programs and insurance companies would need to approve EBP reimbursement, which would likely require input from other federal and state officials and potentially treatment providers; EBP developers can also provide guidance on provider-level standards that would make services eligible for reimbursement. Moreover, federal and state legislators could have important actor roles as they appropriate Medicaid funds, which could limit or expand the amount of support for behavioral health services. One federal official emphasized that they attempt to find funding streams and reimbursement policies that would make EBPs more sustainable and they, “try to teach [organizations] how to fish, not just provide the fish.”

## Discussion

This study synthesized federal and state agency staff's perspectives about improving federal grants for EBP implementation success, including relevant contextual influences, actions, and policy actors. We illustrate how the policy adapted EPIS framework [9] can be used to examine federal funding strategies, a novel area within the growing subfield of policy implementation science. Our findings suggest there are opportunities to improve grant design and implementation during all EPIS phases, with some requiring actions across several phases by various policy actors. The study helps fill the literature gap related to bridging factors, which are understudied [10], and more specifically, the role of federal grant mechanisms.

Themes most relevant to the Exploration phase both related to preference, noting a tendency for grant opportunities to emphasize well-recognized EBPs and neglect the characteristics of low-resourced organizations. Based on guidance for evaluating the quality of evidence for interventions [37, 38], randomized studies are widely accepted as the gold-standard research design and are required for designating “evidence-based” status in many treatment categorization systems [13]. However, officials in our study emphasized selecting treatments based on the *best available* evidence which has implications that other types of evidence (e.g., qualitative data) are still acceptable metrics of evidence when randomized studies are unavailable [13]. Yet, this nuanced difference may not be clear in federal grant language, as one study found that EBPs were required or encouraged in 81% of federal opportunity announcements but a definition of EBPs was only provided in roughly 52% of announcements [13]. Federal agency leaders may consider consciously funding treatment implementation based on the “best available evidence” including results from quasi-experimental and qualitative studies, and/or explicitly defining what they consider an “evidence-based” treatment in announcements. Agency leaders should become educated and cognizant of practices that are less studied but highly-valued and effective in community and cultural contexts (i.e., community-defined evidence). One example is the Community Defined Evidence Project, led by national Latinx and disparities networks, which established processes to identify community-defined practices for populations that remain underrepresented in randomized trials [39]. Regardless of whether the chosen practices are traditionally evidence-based or community-defined, participants emphasized the importance of ongoing fidelity and outcome evaluation to monitor implementation success.

Relatedly, to help treatment organizations with little experience with federal grants, organization leaders may benefit from coaching on strategies to apply to and manage the workload of a federal grant. Federal agencies would first need to identify low-resourced organizations to provide coaching to; one idea is to identify common pitfalls by reviewing unsuccessful applications from organizations and invite the organizations to a grant orientation seminar to fill knowledge gaps. An alternative approach would be to invite organizations that have never applied to a federal grant but serve a high need area to grant orientation seminars. One such example was the 2001 government-backed Faith-Based and Community Initiative (FBCI), which sought to help faith-based and grassroots organizations apply for and complete federally funded social services grants after preference towards awarding previous grant recipients was uncovered [40]. FBCI’s Targeted Capacity Building Program included grant writing support, leadership development and training in outcome measuring and evaluation, which showed some evidence of increased grant applications and awards among organizations in FBCI [38]. Another logistical step that may be difficult is federal funding distribution; federal agencies may consider streamlining that process to simplify grantee communication and begin grant-funded work more quickly. Though such efforts to support organizations would require federal agency funding and personnel time, there would likely be benefits to communities with the highest needs. A valuable future study would be gathering federal funder and treatment organization perspectives about which elements of grant design and application processes should be changed to make grants more accessible for low-resourced organizations.

Several themes in the Preparation and Implementation phases were relevant to the concept of alignment, which refers to the fit between an organization's strategy (e.g., processes and decisions) and objectives [41]. Alignment is an emerging focus in implementation science and has high relevance to federal grants that inherently require interactions at least between federal agencies and awardee organizations [42, 43]. Specifically, the multilevel collaboration theme falls under interorganizational alignment, which refers to the fit between shared objectives and the strategies that different agencies and organizations use [43, 44]. The focus of interorganizational alignment in our findings makes sense as bridging factors, such as grants, link the inner and outer contexts together [10]. Though stronger ongoing collaboration was a discrete theme, any theme that involved individuals from multiple organizations would also fall under interorganizational alignment. For instance, themes about workforce capacity and the tension between fidelity and flexible EBP delivery indicate a need for stronger interorganizational alignment amongst federal or state agencies, awardees, and EBP developers.

Concerns about workforce capacity further underscore the importance of alignment within and between organizations. The high therapist workload in publicly-funded settings increases risk for burnout and turnover, EBPs [43, 45] and when therapists are experiencing financial strain [45, 46]. These studies, coupled with our findings about limited insurance reimbursement for EBPs, bring further attention to the need to increase pay for the behavioral workforce (e.g., providers can be hired full-time in place of precarious contractor positions). Indeed, treatment organizations and providers need guidance to deliver EBPs effectively under real-world service conditions while they grapple with turnover and limited provider time to achieve EBP certification. Though training and technical assistance processes provide opportunities for guidance on EBP delivery, some providers find these difficult or frustrating [47] while others recognize their utility and helpfulness [48]. Training processes could be reframed as opportunities to obtain supervision on the core elements of the EBP but to also share guidance on how to serve their clients, especially those not represented in the trials that helped establish the EBP designation. Furthermore, as EBP implementation without strict adherence to the original model is typical in community settings [49], research on EBP adaptations remains highly valuable. So far, we know that adaptations are common [50], but we have more work to do to better understand how adaptations influence fidelity or outcomes [51]. Involving EBP developers may not only support high-quality EBP delivery [52], but their ongoing involvement could also foster interorganizational alignment [53].

By conceptualizing federal implementation grants as bridging factors, our findings provide ways to address “funding cliffs” when EBP implementation grants end [54]. Participants were not only familiar with challenges during active EBP implementation and sustainment, but also provided potential solutions. One suggestion was to extend grant award periods to allow for more preparation and sustainability planning. Similar findings were found in a study summarizing a 30-year evaluation of SAMHSA’s systems of care grants in Hawaiʻi, which focused on increasing interagency collaboration and integrated approaches to address youth mental health challenges [55]. The perceived benefits of longer grant periods were interrelated to continuity of staff and the promotion of sustainability, as staff who have been trained and have expert knowledge often must find new positions when grants end. Our findings further corroborate how workforce capacity should be a constant consideration when planning and executing federal EBP grants. Additional financial barriers to sustainment included the need to increase insurance coverage of EBPs, especially with public insurance (e.g., Medicaid), which has been identified as a financial strategy for EBP implementation in prior research [2, 22]. Other parallels with our study included the importance of fidelity monitoring during active delivery, and interagency collaboration during the grant process, which we conceptualized as interorganizational alignment [22]. Related to interorganizational alignment, it may be beneficial to set up a network of treatment organizations or state agencies that have been able to successfully procure Medicaid and other insurance reimbursement for EBPs to aid organizations/agencies who have not yet established EBP insurance reimbursement, perhaps through learning collaboratives, trainings or workshops. Though not focused on EBP reimbursement, South Dakota Department of Social Services has provided such technical assistance to help organizations obtain fee-for-service Medicaid reimbursement for Community Health Worker services [56]; such a model would be beneficial for organizations delivering EBPs in the community. In future research, scholars can consider opportunities for quasi-experimental studies comparing EBP implementation outcomes (e.g., sustainment, reach) between systems that have established billing practices for specific EBPs versus those that have not. If aligned with the hypothesis that EBP integration into insurance reimbursement would lead to better implementation outcomes, findings could help influence policy change in support of EBP insurance integration on a wider and potentially systematic scale.

Despite contributions of our study, some limitations should be considered. First, both sample sizes of federal and state agency officials were modest. Relatedly, state agency officials worked in 12 states, and thus their perspectives may not be generalizable to all the United States, tribal governments, or territories. Second, part of our analytic approach was to infer which EPIS phases and policy actors were most relevant to themes. In future work, scholars can consider directly asking government officials who they believe would need to be involved and/or take action to make the recommended changes and when actions should take place. It is possible that there could be more granularity in the policy actors we identified. Third, we did not collect background information about participants to protect privacy. Systematically assessing their previous experiences with federal EBP grants could have informed questions we asked and further contextualized the findings. Lastly, the focus group discussions may have skewed participant perspectives toward issues relevant to youth behavioral health EBPs based on the case example and the participating state agency officials.

## Conclusions

The current study adds to the nascent body of literature characterizing the role of government grants in large-scale EBP implementation efforts [20–22]. To our knowledge, it is the first study focused on improving federal grants to strengthen the potential for EBP implementation success, including key outcomes like reach and sustainment. Gaps persist in how to best improve federal financial support for EBP dissemination which undermines government goals, system efficiency, and the feasibility of high-quality evidence-based treatment delivery. Most notably, EBP reimbursement and workforce turnover have been long-standing challenges that policy actors in our study highlighted [20, 22]. Clarifying when and how various policy actors can take action to support EBP implementation at federal, state, city/county, and treatment organizational levels is crucial to fostering EBP implementation success through federal grant investments.

## Supplementary Information

Additional file 1. Consolidated criteria for reporting qualitative studies (COREQ): 32-item checklist.

Additional file 2. A one-page research summary prior to the focus group that emphasized the role of EBP funding and summarized our findings comparing organization-focused and state-focused SAMHSA grants as a case example.

Additional file 3. Focus group interview guide.

Additional file 4. Summary of contextual influences on EBP implementation success within federal grant context.

## Data Availability

The datasets analyzed for this study are available from the corresponding author upon reasonable request. Depending on the nature of the request, institutional data-sharing agreements may or may not be required.

## Abbreviations

EBP: Evidence Based Practice
SAMHSA: U.S. Substance Abuse and Mental Health Services Administration
A-CRA: Adolescent Community Reinforcement Approach
EPIS: The Exploration, Preparation, Implementation, and Sustainment framework
