# Supplementary material for: Policy actors’ perspectives on improving federal grants to promote the implementation success of evidence-based behavioral health practices
Source: Implement Sci Commun. 2026 Mar 6;7:72. doi: 10.1186/s43058-026-00882-6 (PMC13077903; doi:10.1186/s43058-026-00882-6)
Supplement: Supplementary file 2 — Additional file 2. A one-page research summary prior to the focus group that emphasized the role of EBP funding and summarized our findings comparing organization-focused and state-focused SAMHSA grants as a case example. [file 43058_2026_882_MOESM2_ESM.pdf]

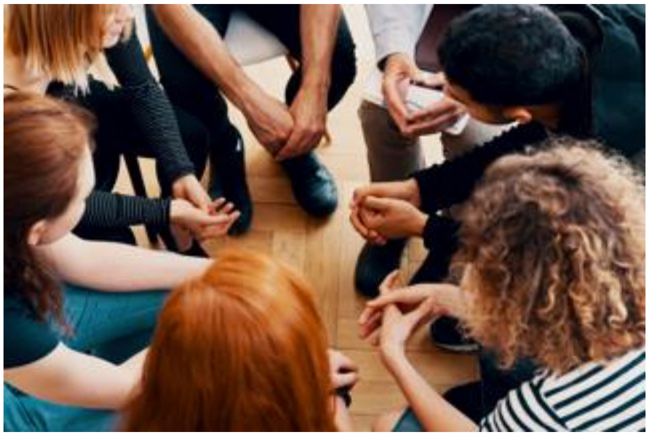

Funding Strategies to Scale Up Evidence-Based Practices in Health and Social Services

How Can State and Federal Agency Officials Help?

The federal initiative “Healthy People 2030” set national objectives for improving health and well-being, across a range of health conditions, behaviors, populations, and settings. We urgently need to address key priority areas—from drug overdose and suicide to HIV and influenza.

Federal spending on high-quality, evidence-based practices (supported by the latest and most rigorous research) in priority health and social services is an investment in offsetting billions of dollars in societal impacts each year. But what is the best way to fund these evidence-based practices?

CASE EXAMPLE

We examined **two types of federal grants** from the Substance Abuse and Mental Health Services Administration (SAMHSA) that supported implementation of the Adolescent-Community Reinforcement Approach (A-CRA). We measured their success by the proportion of providers who became certified in A-CRA.

A-CRA is an evidence-based substance use treatment that helps youth and young adults ages 12–24 replace substance use with adaptive alternative behaviors through cognitive-behavioral and family therapy techniques.

Organization-focused grants

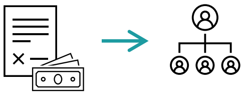

Directly support treatment organizations and providers in implementing A-CRA

State-focused grants

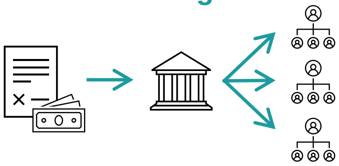

Provide states with funding to guide A-CRA implementation

Organization-focused grants led to more providers getting certified in A-CRA (see Dopp et al., 2023).

- Certification rates were 27 percentage points higher in organization-focused grants.
- Under organization-focused grants, most providers who received training in A-CRA got certified, whereas less than half of providers who trained under state-focused grants were certified.
- We were able to attribute this difference to the grant type, rather than any organizational or state factors.

Federal grants could do more to expand access to evidence-based health and social services.

Under both types of grants, there are opportunities for state and federal agency officials to ensure that evidence-based treatments like A-CRA are made more widely available.

POTENTIAL RECOMMENDATIONS

**Organization-focused grants**	Provide and/or help grantees identify longer-term funding for sustainment (which is difficult after the grant ends).
**State-focused grants**	Provide and/or help state agency officials to develop strategies to navigate challenges (state policies, budget priorities, etc.) to scaling up initiatives at the state level.

State and federal agency officials have valuable knowledge about effective and sustainable funding strategies, as well as the challenges that are likely to arise in allocating grant funding. We welcome your continued input on our recommendations: **RAND Youth Treatment Study, Dr. Alex Dopp, adopp@rand.org**.
