# Supplementary material for: Policy actors’ perspectives on improving federal grants to promote the implementation success of evidence-based behavioral health practices
Source: Implement Sci Commun. 2026 Mar 6;7:72. doi: 10.1186/s43058-026-00882-6 (PMC13077903; doi:10.1186/s43058-026-00882-6)
Supplement: Supplementary file 3 — Additional file 3. Focus group interview guide. [file 43058_2026_882_MOESM3_ESM.pdf]

Reprinted with permission from: Dopp, Alex R., Blanche Wright, Jin Kim, Grace M. Hindmarch, and Sarah B. Hunter, Optimizing Federal Grants to Scale Up Evidence-Based Practices in Health and Social Services: Recommendations from Federal and State Agency Officials. Santa Monica, CA: RAND Corporation, 2025. https://www.rand.org/pubs/research_reports/RRA3627-1.html

APPENDIX C

Focus Group Discussion Guide

Focus Group #1 Guide – Reach Outcomes

Facilitators: ________, ________
Note-taker: ________
Date: ____/____/____
Start Time: ________ End Time: ________
Number of attendees: ________ out of ________
Participant IDs: __________
Type of Focus Group (check one):
☐ State Officials ☐ Federal Officials

VERBAL INFORMED CONSENT

Thank you all for coming today. As a reminder, we are researchers from RAND, a nonprofit organization that conducts nonpartisan research to improve health and well-being. The purpose of today's focus group is to have an in-depth discussion about federal investments in the use of evidence-based practices in health and social services. My name is [NAME] and this is [NAME]; we will lead today's focus group. [NAME] will be taking notes and audio record the session.

The focus group will last about 60 minutes. If you agree to join, the discussion will be audio recorded to make sure we don't miss anything important; the recording will be transcribed with all names removed, and we will destroy the recording once analysis is complete. RAND will keep our discussion confidential, and we will not share any information about your participation with anyone outside of the research team or attribute any of our findings to individuals, although we may quote participants anonymously. RAND cannot guarantee that attendees will maintain confidentiality, but we ask you all to please do not share information about who participated or what was said – it is everyone's collective responsibility to uphold confidentiality of this group.

There is no right answer to any of these questions; we want to hear your thoughts, opinions, and suggestions for policy implications of our work. Your expertise and feedback are invaluable as we continue working to identify the most effective strategies for federal investment into evidence-based practices.

All that said, this focus group is voluntary, and you are under no obligation to participate. You can also discontinue participation at any time or decline to answer specific questions.

If you have any questions, comments, or concerns about the research, please do not hesitate to reach out to us at any time. Furthermore, you can contact RAND's Human Subjects Protection Committee with any concerns; we will put their contact info in the chat. [NOTE-TAKER PASTES INTO THE CHAT: If you have questions about your rights as a research participant or need to report a research-related injury or concern, you can contact the RAND Human Subjects Protection Committee toll-free at (866) 697-5620 or email hspcinfo@rand.org. If you contact the Committee, please reference Study #2020-N0887.]

Does anyone have any questions?

By remaining on this meeting, you are providing your consent to participate and be recorded.

FOCUS GROUP QUESTIONS

[START RECORDING]

To begin, let's have everyone briefly introduce themselves. For now, please just state your name, title, and agency.

As a reminder, we sent each of you a research summary outlining some initial findings from our research. Just to ensure everyone is oriented, we will quickly review that content with you again now. The research summary focuses on the need for funding strategies to scale up evidence-based practices; by evidence-based, we mean practices that have been shown in controlled research to improve clinical outcomes compared to usual practice.

As a case example, we compared two different federal grant initiatives from SAMHSA – the Substance Abuse and Mental Health Services Administration – to disseminate a substance use treatment called the Adolescent Community Reinforcement Approach, or "A-CRA," (also pronounced "ack-ra"). Some grants were made directly to treatment organizations, whereas others were made to state substance

use agencies which then supported multiple treatment organizations; in both grant types, treatment organizations received similar training opportunities and funding amounts. We looked at what percentage of trained clinical staff who achieved "certification," meaning they completed the training process and demonstrated proficiency in A-CRA delivery and/or supervision. Grants awarded directly to treatment organizations had much higher certification rates than grants awarded to state agencies.

Some of you may not have experience with administration or policy focused on youth services and/or substance use services, and that is fine; our focus today is on everyone's broader expertise related to federal grants for implementing evidence-based practices.

2-1. In light of these findings, and your own relevant experiences, what are your views on the use of different federal grant mechanisms to implement evidence-based practices? (As needed, invite participants to share their experiences with federal grants and/or evidence-based practices broadly before answering this question.)

2-2. What are the strengths and limitations of the organization-focused grant model?

2-3. What about the strengths and limitations of the state-focused grant model?

IF NOT COVERED, PROBE:

a. How do these findings compare to your own experiences with similar types of federal grants? Are any parts of the results surprising?
b. In what situations is it more favorable to use organization-focused grants to implement an evidence-based practice? What about state-focused grants?
c. Are there other types of federal grants or funding models that you think are better-suited for implementing evidence-based practices?
d. (FOR ANY OF ABOVE): How does that differ between substance use services vs. other contexts? Between youth services vs. other contexts?

Now, we want to understand your views on the barriers and facilitators to optimizing the use of federal grants to implement evidence-based practices. Optimizing might look like making changes to . . . [Summarize examples of high-priority changes that have been suggested by focus group participants thus far]. Barriers are factors that impede high-priority changes, and facilitators are factors that support those changes. These factors can be at the level of the grant mechanism, the implementation process, the policymakers or agencies involved, and the political and public climate around implementation.

In your view, what are the most critical barriers and facilitators to optimizing how federal grants can best support implementation of evidence-based practices? We would like to hear about factors specific to organization-focused grants and/or state-focused grants, as well as factors more generally relevant to federal funding for evidence-based practices.

PROBE AS NEEDED THROUGHOUT, IF UNCLEAR:

How does that differ between organization-focused vs. state-focused grants?

Substance use services vs. other contexts?

Youth services vs. other contexts?

3-1. What about the broader political and public climate for implementation? This can include factors at the national level, and also factors specific to grant recipients' communities and contexts – such as service recipient and provider factors.

i. *Recipients of the evidence-based practice: characteristics and needs*
ii. *Public opinion and related pressures or needs, including advocacy efforts*
iii. *Service organizations implementing the evidence-based practice: personnel/workforce, leadership, culture, capacity, priorities, partnerships*
iv. *Primary funding sources for service organizations and state agencies*
v. *Partisanship and political support*

3-2. What about factors related to federal agency involved in awarding and administering the grant? This could include things like the culture, priorities, partnerships, and resources of that agency and other relevant parties . . .

i. *Federal agency employees and leadership*
ii. *Federal legislature*
iii. *Federal executive branch*
iv. *Federal laws and policies impacting services, implementation, and/or grants*

3-3. What about factors related to state agencies involved in managing the grant or otherwise supporting grant activities? This could include things like the culture, priorities, partnerships, and resources of that agency and other relevant parties . . .

i. *State agency employees and leadership*
ii. *State legislature*
iii. *State executive branch*
iv. *State laws and policies impacting services, implementation, and/or grants*

3-4. What about factors related to how the grant mechanism is designed and executed by the federal agency? For example . . .

i. *Grant mechanism characteristics, like evidence supporting effectiveness, complexity, cost, relative advantage, etc.*
ii. *Requirements for grant recipients to spend money on certain implementation activities, like training, partnership building, policy development, etc.*
iii. *Fit of grant mechanism, evidence-based practice and implementation context*
iv. *Flexibility to tailor the grant mechanism to the implementation context*

3-5. What about factors related to the process of implementing the evidence-based practice during the grant period? For example . . .

i. *Evidence-based practice characteristics, like the developer or evidence base, regarding distinct cultural groups,*
ii. *Engagement or partnerships with implementation supports, for things like training and technical assistance, implementation planning, or building capacity for data collection/evaluation*

3-6. How can federal agencies help to ensure accountability in the effective use of grant funds toward the implementation of evidence-based practices?

i. *Should certain facilitating factors (or lack of certain barriers) be required of grantees before receiving federal grants to implement evidence-based practices? If yes, which ones?*
ii. *How can recipient outcomes be evaluated or monitored during an award?*
iii. *How can the implementation process (e.g., fidelity) be evaluated or monitored during an award?*
iv. *How can all of this be done in ways that still address health and resource inequities between states and regions?*

Reflecting on everything we discussed today, what do you think is the outlook for using federal grants or other funding mechanisms to implement and sustain evidence-based practices? What are the biggest obstacles and the biggest opportunities in this space?

IF UNCLEAR, PROBE:
How does that differ between substance use services vs. other contexts?
Between youth services vs. other contexts?

Is there anything else you think is important to share that hasn't been discussed?

Thank you for your time. We'll turn off the recorder now.

[STOP RECORDING]

That is the end of the focus group discussion for today. Thank you so much for your time. It was very helpful to hear your perspectives and learn from you.

Does anyone have questions before they sign off?
