# Supplementary material for: Policy actors’ perspectives on improving federal grants to promote the implementation success of evidence-based behavioral health practices
Source: Implement Sci Commun. 2026 Mar 6;7:72. doi: 10.1186/s43058-026-00882-6 (PMC13077903; doi:10.1186/s43058-026-00882-6)
Supplement: Supplementary file 4 — Additional file 4. Summary of contextual influences on EBP implementation success within federal grant context. [file 43058_2026_882_MOESM4_ESM.docx]

Additional File 2. Summary of Contextual Influences on EBP Implementation Success within Federal Grant Context

| **EPIS Domain** | **Code** | **Brief Description** | **Exemplar Quotes** |
| --- | --- | --- | --- |
| **Bridging Factors** | Formal grant arrangements | Features of grant strategies such as complexity, cost, relative advantage over other grant mechanisms, etc. and specifications in requests for proposals, grant application processes (including peer review and funding decisions), and/or awarded grants that influence EBP reach. | “Somehow less at the state implementation than at the individual organization level was a large technical assistance contract where we use the same common instrument, training, both an evaluation for evaluators as well as implementation and coaching and data management ... essentially it was health services research. NIDA funded investigators to do this. SAMHSA did the service delivery grants and we monitored that at our end for the implementation and then had a common contractor that also had NIDA researchers involved as well, sort of the care delivery at the finish line if you will” (facilitator) |
|  | Tailoring grants to context | Ability of funder and/or EBP implementers to tailor grant requirements and activities to the needs of the EBP, organization, community, state, or other contextual factors related to reach. | “I think we lean though towards being less prescriptive in our grants. And if you look at our notices of funding our opportunities, we have very consistent kind of basic language where we ask an applicant to identify an evidence-based practice that they will implement through a given services grant. Talk a little bit about how that evidence-based practice meets the needs of the community they're serving, how it responds to SAMHSA sort of interest as far as that grant program is concerned. We do have a more expansive orientation to evidence-based practice. We talk about culturally defined evidence, practice-based evidence” (facilitator) |
|  | Engaging and partnering with EBP implementers in grant activities | Training and technical assistance, implementation planning, capacity building, and other forms of engagement/partnership with the organizations/individuals implementing the EBP, to maximize reach. | “How important it is for the organization to have a relationship with the state. I'm in a unique position where I started at organization level and then went on to the state and I've had the opportunity to be on both types of funding actually. So, I can definitely see the pros and cons. But what [STATE NAME] did was begin to start it off with that relationship, like it had always been like even upon writing for a federal grant, it was from input from the state. So, even an organization level and then moving forward, and also [STATE NAME] built that infrastructure so that we can sustain, where my position comes from, sustain that workforce turnover that everyone's talking about. So, strategizing that way, building that in and knowing the challenges that are going to come up because we know that they are normal & expected workforce challenges” (facilitator/barrier) |
| **Inner Contexts** | Treatment Organization Personnel | Characteristics of service delivery providers, managers, and leadership that influence their role in EBP reach (especially in cases where grant funds are awarded directly to service org). | “I think champions are huge whenever there's an EBP or an emerging piece. So, if there's a way to support champions within those agencies” (facilitator) |
|  | Treatment Organization Culture and Climate | Characteristics of service delivery organization culture, priorities, communications, resources, etc. that influence their role in EBP reach (especially in cases where grant funds are awarded directly to service org). | “Obviously there was buy in from the organization at the beginning, because it's not easy to apply for a grant. There's a lot of resources and time and effort put into that. So that means that the agency has already bought into that. Sometimes grants do not allow for time. They wanted providers to be selected prior to even having, in the grant solicitation” (facilitator) |
|  | Treatment Organization Policies | Policies and procedures at the service delivery organization that influence their role in EBP reach (especially in cases where grant funds are awarded directly to service org). | N/A |
|  | State Agency Personnel | Characteristics of state agency workers and leaders that influence their role in EBP reach (especially in cases where grant funds are awarded to the state agency). | “So my programs, I've encouraged them if you have any unspent funds. I'm not going to approve you buying a new car or painting an office. But if you're telling me that you want to give somebody a bonus, because either they completed a training or they've met a particular outcome specific to their program, things like that, from my end as a program lead, I'm absolutely approving all of those” (facilitator) |
|  | State Agency Culture and Climate | Characteristics of state agency culture, priorities, communications, resources, etc. that influence their role in EBP reach (especially in cases where grant funds are awarded to the state agency). | “I think this was alluded to is that states really are able to understand the context of where that money can or should be going and also think a little bit more holistically about different funding streams, especially if you're thinking about something like SAMHSA grants. Ideally, and it happens at different extents, states should be thinking about behavioral health modernization and [grants] and opioid litigation and all these things together. And the other thing that I would add, and this is a conversation we're having with states a lot right now is, it takes a certain amount of sophistication to apply for state and federal grants. And unless you kind of take steps to level the playing field, you're going to divert most of the resources to organizations that may not be serving the people that are most at risk. So, states are taking a lot of steps and I'm happy to talk more about it in terms of trying to lower those barriers for BIPOC-led organizations and other entities serving some of the communities that are at risk, of overdose for instance” (barrier/facilitator) |
|  | State Agency Policies | State policies and laws that influence the state agency's role in EBP reach (especially in cases where grant funds are awarded directly to the state agency). | “I think one of the things that came to mind and I don't know if it's here, but one of the things that's been beneficial as we've secured federal funding to support some of our SUD treatment efforts has been to give some funding to the providers to pay for non-Medicaid billable activities, right? So, it's one thing to say, ‘John’ is in treatment, he showed up once a week for 13 weeks for A-CRA and then we're billing for that. But there isn't any cushion to say why I called ‘John’ five times and he said he was going to come today but he didn't show up but the trainer or whoever the clinician was here waiting on him; I had to drop ‘John’ off; or these things that are really-- when we think about young people and things we would say when I try to explain youth SUD to folks and even to the providers, you have to have a philosophy with people what it means to engage young people, right? This isn't somebody who has been using drugs for 20, 30 years, you jacked your life up and now you’re trying to get your life back on track, right? This is a 15, 16, 17 up to 20 year-old saying, I don't think I have a problem, right? So, then how do you engage and encourage and get that kind of person to see that it can benefit from this? But then once again, that time, that effort and that massaging is not Medicaid billable. And so, one of the benefits of federal funding that we've been able to use at least has been to support some of those outreach, engagement, and follow-up activities” (barrier/facilitator) |
| **Outer context** | Federal Agency Personnel | Characteristics of awarding federal agency workers and leaders that influence their role in EBP reach during the grant period. | N/A |
|  | Federal Agency Culture/Climate | Characteristics of awarding federal agency culture, priorities, communications, resources, etc. that influence their role in EBP reach during the grant period. | “Yeah, so I just want to say that as flawed as the federal funding mechanisms are in the ways that we've discussed the challenges. I don't see any other push really any of substantive, you know, there are some foundation work being done, but really the federal emphasis on evidence-based practice is still the main way they are occurring out there. So, I would take all of the flaws we currently have over nothing” (barrier/facilitator) |
|  | Federal Agency Policies | Federal policies and laws that influence the awarding federal agency's role in EBP reach during the grant period. | N/A |
|  | State Executive and Legislative Politics | Activities of the state executive branch (e.g., governor's office) and/or legislature (e.g., representatives, senate) that impact EBP reach, directly or indirectly; includes executive orders and laws but also hearings, partisanship/political support, etc. | “And thankfully, we are now a Medicaid expansion state. So I know how you feel about-- back in the day, because it's like, I don't even know how to manage the things that other states are able to manage right now. And all we have CCBHCs, Certified Behavioral Health Clinics, and it covers every county. So that has been hugely supportive of the work that we're doing” (facilitator) |
|  | Federal Executive and Legislative Politics | Activities of the federal executive branch (e.g., President's office) and/or Congress that impact EBP reach, directly or indirectly; includes executive orders and laws but also hearings, partisanship/political support, etc. | N/A |
|  | Public Opinion and Pressure | Activities of the general public that influence policies and political decision-making related to EBP reach, directly or indirectly; includes advocacy and lobbying, constituent polls, but also informal interactions such as calls and letters to elected officials. | N/A |
|  | Recipient and Community Factors | Characteristics and needs of EBP recipients (individuals and/or broader communities) that impact reach, especially as related to the EBP's responsiveness to those characteristics and needs. | “But what has been beautiful for us as - unfortunately, through COVID, [A-CRA purveyor] was amenable to create the virtual training option and we still continue that. And so that's been a huge save on financial costs for not only for the training, but for some of our agencies, because [STATE NAME] is such a huge rural state. It costs money to send people to training and lodging and things like that” (barrier/facilitator) |
|  | Professional and Workforce Factors | Characteristics of the broader professions involved in implementing and delivering the EBP that impact reach, such as requirements for professional licensure or accreditation, accepted standards of practice in the field, continuing education processes, trends and issues within the workforce, etc. | “So, we have that in [STATE NAME] but it's the incentive I can't give as much as I would like to. So, focusing on before they hit-- become a clinician.    Interviewer: Can I ask? And if this would take a long time to explain you can defer it, but what is the mechanism for that incentive? Is it like a bonus for working in certain settings or?    Interviewee: No, it's a requirement they take part in the training and certification just like any clinician. And they are placed at a provider that has that supervision available and the clients and they have to achieve certification. We know that is the gold standard” (facilitator) |
|  | Primary Funding Sources for the EBP | Characteristics of primary funding sources for service organizations and state agencies in their EBP implementation/ delivery efforts, such as availability, feasibility, and effectiveness. | “I think helping grantees to be able to do Medicaid billing is a big one and many of them are intimidated by that and don't want to tackle it. [Audio cuts] those that are being discussed are Medicaid billable, that really is the way to sustain them... guess one thing I would add is that Medicaid is important and also some of these have been able to tap on commercial insurance. So, just increasing capacity to give insurances will be one important thing” (barrier/facilitator). |
| **Innovation** | EBP Factors | Characteristics of selected EBPs (including its developer/training model) such as learnability, effectiveness, cultural responsiveness, etc. that impact reach. | “We do have specific rates for very specific evidence-based interventions and they're generally enhanced rates - so they're a little bit higher. A good example would be for TFCBT, we have, regular outpatient rate, then we have an enhanced rate” (facilitator) |
|  | Evidence base | Evidence supporting the EBP, grant type, etc. is described in ways that relate to reach (e.g., increased widespread buy-in). | “We find that states are gravitating to a few programs that have much longer track records and higher levels of evidence and are less interested… programs that are less well known… they're going off for the name brand… and have been less interested in developing additional models or evidence for other models” (barrier/facilitator) |
|  | Fit with Context | Fit between the EBP, grant strategy, and the needs of the organization, community/recipients, state, or other contextual factors related to reach. | “I think sort of spurred by COVID, there's growing realization that community health workers, community-based organizations are really critical partners when it comes to delivering community-based health services and related social needs as states are beginning to think about that more and more in a Medicaid context” (facilitator) |

*Note*. Parentheticals indicate whether quote characterizes a facilitator or barrier. To preserve participant confidentiality, we do not specify if quotes were from state or federal officials. A-CRA = Adolescent Community Reinforcement Approach. EPIS = the Exploration, Preparation, Implementation, and Sustainment framework. EBP = Evidence Based Practice. SAMHSA = U.S. Substance Abuse and Mental Health Services Administration.
